# Evaluation of Functional Outcomes and OCT-Biomarkers after Intravitreal Dexamethasone Implant for Postoperative Cystoid Macular Edema in Vitrectomized Eyes

**DOI:** 10.1155/2020/3946531

**Published:** 2020-04-28

**Authors:** Sigrid Freissinger, Efstathios Vounotrypidis, Armin Wolf, Karsten U. Kortuem, Mehdi Shajari, Filippos Sakkias, Tina Herold, Siegfried G. Priglinger, Wolfgang J. Mayer

**Affiliations:** Department of Ophthalmology, Ludwig-Maximilians-University, Munich, Germany

## Abstract

**Purpose:**

To evaluate the efficacy of dexamethasone implant (DEX) for the treatment of postoperative cystoid macular edema (PCME) in vitrectomized eyes and to investigate visual and morphological OCT predictive factors.

**Methods:**

In this retrospective study, eyes with PCME after vitrectomy were treated with at least one DEX injection and were observed over 12 months. Indications for surgery were epiretinal membrane (ERM) or rhegmatogenous retinal detachment (RRD) without macular involvement. Prior treatments, if any, were noted. Best corrected visual acuity (BCVA), central foveal thickness (CFT), and OCT morphology including the presence of intraretinal cysts/fluid or subretinal fluid (IRF/SRF) and ellipsoid zone (EZ) continuity were evaluated. Correlations between OCT measures and visual outcomes were analyzed by the generalized estimating equations procedure.

**Results:**

Forty-six eyes with ERM and 15 eyes with RRD were enrolled. The ERM group was more likely to gain BCVA than RRD (odds ratio (OR), 1.168; 95% confidence interval (CI), 1.003–1.360; *p*=0.046). The absence of SRF (OR, 0.860; 95% CI, 0.743–0.995; *p*=0.043) was predictive of worse BCVA, whereas the integrity of EZ (OR, 1.094; 95% CI, 0.951–1.257; *p*=0.209) or naïve status (OR, 0.946; 95% CI, 0.871–1.137, *p*=0.853) was not. Eyes with a worse baseline BCVA were more likely to gain >1 line after 12 months (OR, 1.485; 95% CI, 1.171–1.884; *p*=0.001).

**Conclusion:**

The efficacy of the treatment of PCME in vitrectomized eyes seems to be affected by baseline BCVA, the absence of SRF, and the indication for surgery. Naïve status appears not to play any significant role in the prediction of BCVA. This trial is registered with DRKS00018955.

## 1. Introduction

Postoperative cystoid macular edema (PCME) is one of the major reasons for visual impairment after cataract surgery, vitrectomy, or combined phacovitrectomy and usually occurs within 4 to 12 weeks [1]. In most of the cases, it resolves without treatment, but if no resorption occurs, structural changes of the retinal layers may lead to irreversible visual deterioration [2]. The pathomechanism of PCME is considered to be multifactorial [3–5], and to date, intraoperative complications (i.e., posterior capsule rupture), epiretinal membrane, vein occlusion, topical prostaglandin therapy, diabetes, and uveitis have been identified as risk factors [6, 7].

Treatment of PCME consists of topical nonsteroidal anti-inflammatory drugs (NSAIDs) and periocular, systemic, or intravitreal cortisone [6, 8]. In general, topical eye drops are applied as the initial treatment, followed by periocular and intravitreal corticosteroids [6], as they offer several advantages compared with systemic corticosteroids [9, 10]. However, their disadvantage is their short-lasting effect [11]. The faster clearance in vitrectomized eyes also reduces the effect of intravitreal steroids in comparison with nonvitrectomized eyes [12].

The longer-lasting single sustained-release dexamethasone intravitreal implant (Ozurde®, Allergan Inc., Irvine, CA, USA, and Allergan Pharmaceuticals, Ireland) is used to achieve steroidal shielding in PCME cases [13, 14]. It is maintained up to 180 days after administration [15, 16]; its effect lasts up to six months after injection, even in vitrectomized eyes [17, 18], and its efficacy and unaffected pharmacokinetic has been demonstrated in experimental and clinical trials in nonvitrectomized and vitrectomized eyes [15, 19–21].

Until now, dexamethasone intravitreal implant (DEX) has been widely used for the treatment of diabetic macular edema, noninfectious uveitis, and secondary macular edema after retinal vein occlusion [20, 22–24]. However, during the last few years, it has been more favorably applied for the treatment of PCME, regardless of preoperative diagnosis [10, 25–27].

The efficacy of DEX in the treatment of PCME after vitrectomy for ERM has been reported in several case studies [27–29] and in two large retrospective studies [30–32], whereas only two studies have evaluated DEX after vitrectomy for RRD repair [31, 33]. However, no study has as yet evaluated any visual or morphological OCT biomarkers for their predictive value in the treatment of PCME in vitrectomized eyes.

This retrospective study was conducted to evaluate the long-term safety and efficacy of DEX for the treatment of PCME in vitrectomized eyes after surgery for ERM removal or RRD repair. Furthermore, various parameters were evaluated for their influence in visual outcome one year after the first DEX injection. OCT morphology including the presence of subretinal fluid (SRF), intraretinal fluid (IRF), ellipsoid zone (EZ) continuity, central retinal thickness (CRT), prior treatments, duration between vitrectomy and onset of PCME, duration between vitrectomy and initial DEX injection, lens status at time of vitrectomy, IOP-increase, and PCME recurrence and persistence were evaluated over a period of 12 months.

## 2. Materials and Methods

### 2.1. Design

A retrospective longitudinal case series was established at the Department of Ophthalmology in Ludwig Maximilians University, Munich. The study was approved by the institutional review board of the Department of Ophthalmology, Ludwig Maximilians University, Munich, and adhered to the tenets of the Declaration of Helsinki (registration trial: DRKS00018955). Based on an electronic database warehouse, all vitrectomized eyes that developed PCME after surgery including treatment with at least one DEX injection and a minimum follow-up period of 12 months were selected. The indication for surgery was restricted to epiretinal membrane (ERM) or rhegmatogenous retinal detachment (RRD) without macular involvement. All eyes were pseudophakic after surgery and had an in-the-bag intraocular lens. Postoperative cystoid macular edema was diagnosed clinically and by means of spectral-domain optical coherence tomography (OCT, Spectralis, Heidelberg Engineering, Heidelberg, Germany).

Patients with previous vitrectomy, residual ERM, postoperative retinal detachment, or further intraocular surgeries during the follow-up period or topical therapy with prostaglandins were excluded. Additionally, eyes with any underlying retinal pathology that could affect the development of a cystoid macular edema, such as previous ocular trauma, age-related macular degeneration (AMD), diabetes, retinal vascular occlusion (RVO), vitreous hemorrhage, uveitis, silicon oil filling, or proliferative vitreoretinopathy, were excluded.

### 2.2. Surgical Procedure

Pars plana vitrectomy (23 gauge) included core vitrectomy, posterior vitrectomy, and vitreous base shaving. Internal limiting membrane peeling by using dye (brilliant blue) and Eckart forceps was performed in all cases. PFCL was used only in RRD cases. Balanced salt solution (BSS) was injected as a tamponade for ERM cases, whereas a gas tamponade (C2F6-15%) was used for RRD repair. Moreover, laser coagulation was always performed after RRD surgery and when required in the other cases. Postoperative treatment included combined antibiotics and steroid eye drops 4 times a day, reduced by 1 eye drop every week.

The injection of the DEX implant was administered under local anesthesia (topical lidocaine) and sterile conditions according to the manufacturer's recommendations by using the provided 22-gauge injecting applicator. Injection was performed in the inferotemporal or superotemporal quadrant.

The implant was injected in the treatment naïve eyes and in cases with PCME that had previously been treated with parabulbous steroid injections (40 mg triamcinolone) and/or topical nonsteroidal anti-inflammatory eye drops (nepafenac 1 mg/ml three times daily).

### 2.3. Examinations

Several variables were recorded and analyzed including patient demographics, lens status at surgery, indication for surgery, time between surgery and onset of PCME, time between surgery and first DEX injection, prior treatments, and number of DEX implants within the follow-up period.

Best corrected visual acuity (BCVA), optical coherence tomography (OCT, Spectralis, Heidelberg Engineering GmbH), clinical examination including fundus biomicroscopy, and intraocular pressure (IOP) measured by Goldmann applanation tonometry were assessed prior to the initial DEX injection and one year after the first DEX injection. BCVA was assessed with Snellen optotypes. Central retinal thickness (CRT) involving the central foveal area of the Early Treatment Diabetic Retinopathy Study (ETDRS) macular grid was obtained. The segmentation of the retinal thickness between Bruch's membrane and the retinal nerve fiber layer had been previously noted and, if necessary, manually adjusted. OCT-morphological changes including the presence of intraretinal cysts/fluid (IRF), subretinal fluid (SRF), and EZ continuity were documented by an experienced physician (SF) and analyzed.

The eyes were separated into two groups with regard to the indication for surgery (ERM or RRD) or prior treatment to the first DEX injection (naïve or previously treated). Further parameters such as recurrence or persistence of PCME were evaluated. Recurrence of PCME was defined as an increase of CRT >400 microns and the presence of IRF and/or SRF. The persistence of PCME was defined as no decrease of CRT compared with the time of initial DEX injection and the presence of IRF and/or SRF, despite multiple DEX injections.

Study-specific adverse events of the single sustained dexamethasone implant were reported. In particular, a patient with an elevated intraocular pressure (>25 mmHg) after injection with the necessary prescription of IOP-lowering therapy was assessed as a steroid responder.

### 2.4. Statistical Analysis

Statistical analysis was performed by using SPSS Statistics 24 (IBM, Armonk, NY, USA). Normal distribution was tested with the Kolmogorov–Smirnov test. Parametrical and nonparametrical tests were performed for BCVA and OCT data between the baseline and final follow-up. Data are presented as the mean ± standard deviation (range). A value of *p* < 0.05 was considered statistically significant.

The generalized estimating equation (GEE) procedure was used to calculate differences in functional treatment response between naïve and refractory patients and included baseline BCVA (prior to the initial DEX injection) as a covariate. An improvement of BCVA of more than 0.1 log MAR to baseline was classified as visual gain, a change within ±0.1 log MAR as no change, and a loss of more than 0.1 log MAR as visual loss.

The GEE model for outcome at 12 months was run by testing the following predictive factors at baseline: (1) BCVA; (2) indication for surgery; (3) lens status; (4) naïve status; (5) presence of IRF; (6) presence of SRF; (7) EZ continuity; (8) duration between surgery and onset of PCME; (9) duration between surgery and first DEX injection. Predictors were entered into the model and kept within it if the *p* value was less than 0.10. The final GEE model was used to calculate the odds ratios (ORs) and their 95% confidence intervals (CIs), with a change of 0.1 log MAR in baseline BCVA being considered as a standard unit of change. Values are presented as the mean ± standard deviation (95% CI).

## 3. Results

A total of 61 eyes (30 right/31 left) of 61 patients (34 women, 27 men) were included in the study. The mean age of the patients was 64.8 ± 10.2 years (35–90). Indication for surgery was ERM in 46 eyes (24 phakic, 22 pseudophakic) and RRD in 15 eyes (6 phakic, 9 pseudophakic).

Table 1 presents the demographic data, indication for surgery, lens status, previously treated eyes, mean number of injections, mean duration between surgery and onset of PCME, and mean duration between the diagnosis of PCME and first DEX injection.

**Figure 1:**
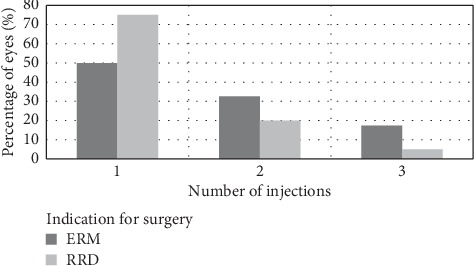
Percentage of eyes that received 1, 2, or 3 DEX injections with regard to indication for surgery. The percentage of eyes progressively declined with increasing number of injections for both indications for surgery. ERM: epiretinal membrane, RRD: rhegmatogenous retinal detachment, and DEX: dexamethasone implant.

**Figure 2:**
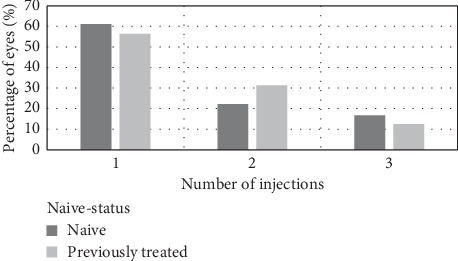
Percentage of eyes that received 1, 2, or 3 DEX injections with regard to naïve status. The percentage of eyes progressively declined with increasing number of injections regardless of naïve status.

The mean number of DEX injections was 1.57 ± 0.7. The number of eyes that required a second or a third injection progressively declined during the follow-up period, regardless of the indication for surgery or naïve status. The percentage of eyes that received 1, 2, or 3 injections, with regard to the indication for surgery, is demonstrated in Figure 1 and with regard to naive status is shown in Figure 2.

In total, 43 eyes (70.5%) were treated prior to the first DEX injection, whereas 18 eyes (29.5%) were initially treated with DEX. Thirty-three eyes (23 ERM and 10 RRD) had previously been treated with parabulbous triamcinolone injection, and 10 eyes (8 ERM and 2 RRD) with topical nonsteroidal anti-inflammatory eye drops. In ten cases previously treated, PCME was unresponsive (6 ERM and 4 RRD), and in the rest of 33 cases, PCME was recurrent. Twenty-one patients were receiving antihypertensive medication at the time of the DEX injection, but no retinal signs of hypertension were present at any time during the study.

### 3.1. Recurrence, Persistence, and OCT Morphology of PCME

Of the 35 eyes that received one DEX injection (23 ERM and 12 RRD), four eyes showed PCME after 12 months. Two eyes presented a recurrence (1 ERM and 1 RRD), with massive IRF, increase of CRT, and visual impairment, whereas two eyes (1 ERM and 1 RRD) had a persisting PCME without any change of BCVA and a CRT >510 microns. The eyes with persisting PCME showed a completely disrupted EZ during the last follow-up examination.

Of the 17 eyes that received two DEX injections (15 ERM and 2 RRD), four presented a PCME at the last follow-up. A recurrence was diagnosed in three cases (3 ERM). In one eye, a persisting PCME was observed with a CRT of 400 microns and no change of BCVA (ERM).

Similarly, of the nine eyes (8 ERM and 1 RRD) that received three DEX injections, three eyes showed a recurrence (2 ERM and 1 RRD) and one eye showed a persisting PCME with a completely disrupted EZ (ERM).

At the last follow-up examination, 49 eyes (80.3%) showed no PCME. Of the 19 eyes that presented with subretinal fluid prior to the first DEX injection, only five (8.2%) showed a subretinal fluid at the last follow-up examination (*p*=0.004, Mc-Nemar test). Prior to the first DEX injection, EZ was intact in 12 eyes (19.7%), partially disrupted in 31 eyes (50.8%), and completely disrupted in 18 eyes (29.5%). At the last follow-up, 31 eyes (50.8%) presented with an intact EZ, 23 eyes (37.7%) with a partially disrupted EZ, and 7 eyes (11.5%) with a completely disrupted EZ (*p*=0.001, Cochran's *Q* test).

### 3.2. Visual Acuity and Central Retinal Thickness

In the ERM group, BCVA improved from 0.69 ± 0.21 (0.3–1.1) to 0.46 ± 0.29 (0.1–1.1) log MAR (*p* < 0.0001, Wilcoxon test) and CRT decreased from 512.3 ± 125 (285–740) to 369.6 ± 108.3 *μ*m (225–733) (*p* < 0.0001, Wilcoxon test). The improvement of BCVA in the RRD group from 0.58 ± 0.17 (0.3–0.8) to 0.53 ± 0.34 (0–1.2) log MAR was not statistically significant (*p*=0.542, paired *t*-test), although CRT decreased from 457.1 ± 85.2 (302–639) to 377.6 ± 99.6 *μ*m (245–566) (*p*=0.016, paired *t*-test).  Table 2 demonstrates BCVA and CRT at baseline and after one year with regard to indication for surgery and prior treatments.

**Figure 3:**
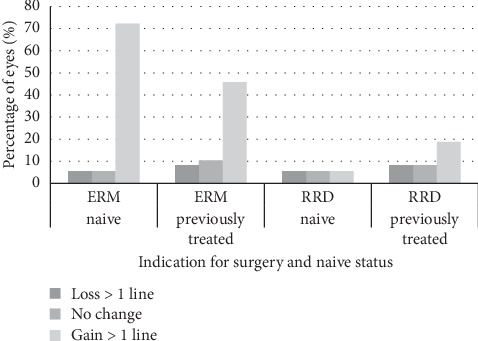
Percentage of eyes that lost or gained >1 line of CDVA or remained stable with regard to naïve status and indication for surgery. Naïve ERM seems more likely to gain CDVA and less likely to lose CDVA or remain stable in comparison with previously treated eyes. On the other hand, RRD eyes seem more likely to gain CDVA, if previously treated. ERM: epiretinal membrane, RRD: rhegmatogenous retinal detachment, and CDVA: corrected distance visual acuity.

The percentage of eyes that showed a visual gain, loss, or no change with regard to diagnosis and prior treatment is demonstrated in Figure 3 for all eyes.

### 3.3. Predictive Factors

The results of the GEE with regard to the odd ratios of predictors of good functional treatment response after 12 months are presented in Table 3. Indication for surgery, absence of SRF, and baseline BCVA seem to have a significant effect on the final outcome, whereas lens status at surgery, EZ integrity, presence of IRF, and number of DEX injections do not seem to influence the improvement of BCVA one year after the initial DEX injection. The odds gain in BCVA was increased in eyes that underwent surgery because of ERM compared with RRD (OR, 1.168; 95% CI, 1.003–1.360; *p*=0.046) and in eyes with a worse baseline BCVA (OR, 1.485; 95% CI, 1.171–1.884; *p*=0.001). Absence of SRF at baseline examination was a predictive factor for loss in BCVA (OR, 0.860; 95% CI, 0.743–0.995; *p*=0.043). In a subanalysis, in which eyes were excluded that showed no response to DEX, the absence of SRF seemed again to be a predictive factor of a worse visual outcome (OR, 0.848; 95% CI, 0.759–0.948; *p*=0.004), whereas intact EZ continuity was a predictive factor of a better final BCVA (OR, 1.137; 95% CI, 1.015–1.273; *p*=0.027).

### 3.4. Adverse Events

No serious adverse events, such as vitreous hemorrhage, endophthalmitis, or dislocation of the implant in the anterior chamber, were associated with dexamethasone injection. An elevated intraocular pressure (IOP), defined as pressure over 25 mmHg measured at any time after DEX injection, was assessed in 18% of the cases. These patients received topical IOP lowering treatment, but none required additional glaucoma surgery.

## 4. Discussion

The efficacy of DEX in the treatment of PCME has been shown in several studies after cataract surgery [13, 31, 34], in two large cohorts in vitrectomized eyes after surgery for various underlying pathologies [30, 31], and, recently, in a small cohort after surgery for RRD with macular involvement [33]. However, most of these studies have focused on the development of CRT and BCVA after DEX injection and its adverse effects. The EPISODIC-2 investigation was the first study that evaluated predictive factors, such as preoperative existing risk factors, naïve status, and age, with regard to a better visual outcome after DEX treatment. Recently, one study has focused on OCT biomarkers for predicting the functional outcome at two and four months after one DEX injection for the treatment of diabetic macular edema [35].

The pharmacokinetics of the 0.7 mg DEX implant is similar in vitrectomized and nonvitrectomized eyes [15, 18]. DEX improves visual acuity, decreases central foveal thickness, reduces the functional and anatomical recurrence of PCME after cataract surgery in nonvitrectomized eyes [13, 31], and also shows positive results in the treatment of PCME in vitrectomized eyes [21, 26, 27, 29, 36, 37] without any increased risk of adverse effects [20].

The results of our study reveal the positive functional and morphological effects of DEX in vitrectomized eyes after surgery for ERM or RRD. BCVA improved in 76.1% of the ERM eyes and in 46.7% of RRD eyes. This was combined with a decrease of CRT at the last follow-up. The improvements in both BCVA and CRT confirm the positive effect of DEX in the treatment of PCME in vitrectomized eyes, as previously reported in several studies [29–31, 33, 35].

Interestingly, the mean number of DEX injections in our study reduced progressively over the follow-up period reflecting the efficacy of DEX, regardless of the indication for surgery. These results are in accordance with the study of Hattenbach et al. [30], in which 17 of 39 eyes required a second DEX injection and 8 of 17, a third treatment. However, in that study [30], only 6 eyes had a longer follow-up (>200 days), no improvement of CRT was noted, and only a slight improvement of BCVA was reported. Furthermore, three of the eyes were later treated with fluocinolone acetate implant. No other information was provided with regard to OCT morphology prior to the DEX injection in their investigation.

In our study, the mean number of required DEX injections did not seem to be affected by naïve status, and this status did not seem to increase the probability of a better final visual outcome. In contrast, in the EPISODIC-2, naïve eyes were reported only to need one DEX injection during the follow-up period [31]. However, EPISODIC-2 study involved 31 vitrectomized eyes (23 ERM and 8 RRD), and only 14 out of 100 enrolled eyes were naïve. Additionally, no information was provided regarding the surgery type of the naïve eyes. Nevertheless, with regard to the hypothesis of the EPISODIC-2 study, namely, that the kind of surgery responsible for PCME seems to be an important factor, the results of our study also indicate that surgery plays an important role in the final visual outcome after DEX treatment for PCME. However, we could find no influence of the preoperative lens status on the final visual outcome. Furthermore, the early (<3 months) or late (>3 months) onset of PCME after surgery and prompt (within 6 months after surgery) or delayed (after 6 months after surgery) treatment with DEX injection did not seem to have any predictive value with respect to the final functional outcome. In the ERM group, the mean BCVA improved the most in eyes that received one injection and progressively decreased with an increasing number of injections. However, in the RRD group, the mean BCVA showed greater improvement in eyes that received two injections. Interestingly, the mean BCVA improved the most in naïve eyes after 2 injections and in pretreated eyes after three injections.

To date, the efficacy of DEX in vitrectomized eyes for the treatment of recalcitrant PCME after RRD repair has been investigated in one retrospective study that included 17 eyes [33]. However, major differences exist between that previous study and our cohort. Thanos et al. evaluated patients with recalcitrant PCME, and all eyes had RRD with macular involvement; most of them were complex cases including PVR (12/17) and silicone oil use (8/17), and some cases underwent vitrectomy combined with scleral buckling [33]. In our cohort, none of the included eyes had macular involvement, all underwent only one surgery during the follow-up period, no silicon oil was used, and no scleral buckling was performed. Therefore, in our cohort, the mean BCVA at the baseline was better, and the CRT was lower. Similarly, the small number of patients that developed an elevated IOP (2 eyes) in our study could be treated adequately with topical IOP-lowering medication. The average of 4 injections over the follow-up period in the study of Thanos et al. reflects the complexity of the enrolled cases in comparison with the 1.3 injections of our study over the 12-month follow-up period. In our cohort, 12 eyes received only one DEX injection, 2 eyes received two injections, and 1 eye received three injections, indicating the high efficacy of DEX in majority of the cases.

Up to now, only one study has evaluated the predictive value of morphological structures of OCT after treatment with DEX for DME [35]. To our knowledge, our study is the first to evaluate the predictive value of a variety of factors, such as OCT biomarkers, indication for surgery, lens status, and baseline BCVA in the treatment of PCME with DEX in vitrectomized eyes. Subretinal fluid was present in 19 eyes at baseline and only in 5 eyes after 12 months, whereas EZ continuity was intact in 14 eyes at baseline and in 31 eyes one year later. Interestingly, the absence of SRF and a better baseline BCVA seemed to be predictive factors of a worse visual outcome.

The only significant adverse effect that has been reported after DEX injections is the elevation of IOP. In our study, 18% (11/61) of the patients showed a raise of IOP over 25 mmHg and needed topical therapy. Most of the studies published to date record a similar prevalence of IOP elevation after DEX implantation, whereas Chin et al. have reported a higher percentage (26.9%) [38]. We have observed no luxation of DEX in the anterior chamber, although all the patients of our cohort were pseudophakic (PC-IOL in the bag) and vitrectomized at the time of the injections.

In this study, we have investigated the efficacy of DEX in the treatment of PCME in vitrectomized eyes after ERM removal or RRD repair. Our results suggest that DEX should be considered as the first-line treatment option. The improvement of BCVA, the reduction of CRT, and the low incidence of IOP elevation strengthen the previously published data concerning the safety and efficacy of DEX in such cases [25]. Furthermore, the analysis of the morphological parameters of the OCT reveals the structural effect of DEX on the retinal layers and provides the predictive value of several variables on the final visual outcome. The decreasing need for further DEX injections over the follow-up time and the high mean BCVA improvement after the first DEX injection, especially in ERM cases, support the idea that DEX should be considered as the first-line therapy in the treatment of PCME. Furthermore, naïve status and lens status at vitrectomy do not seem to play a crucial role in the functional outcome.

The retrospective design of this study is its major limitation. Additionally, the nonequality of the two different groups (ERM and RRD) can be considered as a disadvantage. A further drawback is the fact that naïve eyes were only 29.5% of the cohort, whereas the remaining eyes had been previously treated and some of them were unresponsive. However, all previously treated eyes had experienced the same treatment, which included either parabulbous steroids, the same NSAID eye drops (nepafenac 1 mg/ml 3x daily), or a combination of them, offering a high homogeneity in the previously treated eyes. Moreover, we followed up all patients over a period of 12 months, evaluated BCVA, CRT, IOP elevation, lens status, and OCT biomarkers and recorded the odds ratios and predictive factors of the better visual outcome after treatment with DEX. We enrolled only uncomplicated patients who underwent one surgery during the follow-up time, thereby excluding other factors that might play a significant role in the development of PCME after vitrectomy.

In conclusion, this study demonstrates the efficacy and safety of DEX in the treatment of PCME in vitrectomized eyes after ERM removal or RRD repair and presents factors that have a predictive value for better functional outcome one year after the first DEX injection. The integrity of the EZ layer and the lower baseline BCVA seem to be predictive factors for better visual acuity, whereas the absence of SRF appears to be a predictive factor of worse final visual acuity. Naïve status and lens status at vitrectomy do not seem to play a significant role in that respect. Further studies with longer follow-up and a wider spectrum of diagnosis may reveal further predictive factors. Moreover, although an association between ERM, CME prior to vitrectomy, and the higher prevalence of PCME is known [1, 39], the ERM stage and OCT morphology at the time of vitrectomy should be investigated for their predictive value on the final visual outcome.

## Figures and Tables

**Table 1:** Demographic data, lens status, prior treatments, mean number of injections, mean duration between surgery and onset of PCME, and between the diagnosis of PCME and first DEX injection in all eyes, and with regard to the indication for surgery.

Parameter	All eyes	ERM	RRD
Mean age ± SD, years (range)	64.8 ± 10.2 (35–90)	66.2 ± 10.5 (35–90)	60.5 ± 8.0 (42–75)

Sex, *n* (%)			
Male	27 (44.3)	19 (31.1)	8 (13.1)
Female	34 (55.7)	27 (44.3)	7 (11.5)

Laterality, *n* (%)			
Right	30 (49)	23 (37.7)	7 (11.5)
Left	31 (51)	23 (37.7)	8 (13.1)

Lens status at surgery, *n* (%)			
Phakic	27 (44.3)	24 (39.3)	3 (4.9)
Pseudophakic	34 (55.7)	22 (36.1)	12 (19.7)

Previous treatment, *n* (%)	43 (70.5)	31 (50.8)	12 (19.7)
Topical	11 (18)	9 (14.7)	2 (3.3)
Parabulbous triamcinolone	33 (54.1)	23 (37.7)	10 (16.4)
Naive	18 (29.5)	15 (24.6)	3 (4.9)

Mean number of injections, *n* ± SD	1.57 ± 0.7	1.67 ± 0.8	1.3 ± 0.6
1 injection, *n* (%)	35 (57.4)	23 (37.7)	12 (19.7)
2 injections, *n* (%)	17 (27.9)	15 (24.6)	2 (3.3)
3 injections, *n* (%)	9 (14.7)	8 (13.1)	1 (1.6)

Mean duration between surgery and onset of PCME (months)	3.4 ± 3.1 (0–12)	3.2 ± 3.2 (0–12)	4.1 ± 2.8 (1–12)

Mean duration between the diagnosis of PCME and first DEX (months)	7.7 ± 12.9 (0–44)	6.5 ± 11.1 (0–44)	11.5 ± 17.5 (0–18)

ERM: epiretinal membrane; RRD: rhegmatogenous retinal detachment; SD: standard deviation; PCME: postoperative cystoid macular edema; DEX: dexamethasone implant.

**Table 2:** CDVA (log MAR) and CRT (*μ*m) as mean ± standard deviation (range) at baseline and after 12 months with regard to naïve status and indication for surgery.

Parameter	All eyes		ERM		RRD	
	Naïve (*n* = 18)	Pretreated (*n* = 43)	Naïve (*n* = 15)	Pretreated (*n* = 31)	Naïve (*n* = 3)	Pretreated (*n* = 12)
CDVA at baseline	0.82 ± 0.32 (0.2–1.3)	0.55 ± 0.27 (0.1–1.1)	0.87 ± 0.30 (0.4–1.3)	0.55 ± 0.28 (0.1–1.2)	0.57 ± 0.4 (0.2–1.0)	0.54 ± 0.25 (0.2–1.0)
CDVA at 12M	0.59 ± 0.33 (0.1–1.2)	0.43 ± 0.28 (0–1.2)	0.57 ± 0.33 (0.1–1.1)	0.41 ± 0.25 (0.1–1.0)	0.70 ± 0.36 (0.3–1.0)	0.49 ± 0.35 (0–1.2)
*p* value	*0.008* ^*∗*^	*0.003* ^*∗*^	*<0.0001* ^*†*^	*0.004* ^*∗*^	0.655^*∗*^	0.410^†^
CRT at baseline	472.4 ± 129.4 (285–677)	507.2 ± 114.9 (346–645)	485.9 ± 133.3 (285–677)	525.1 ± 121.0 (350–645)	405.0 ± 98.8 (302–499)	460.9 ± 85.2 (346–639)
CRT at 12M	380.2 ± 109.9 (225–660)	365.5 ± 102.5 (243–733)	377.6 ± 104.3 (225–660)	365.7 ± 111.7 (243–733)	393.3 ± 161.9 (245–566)	365.1 ± 78.1 (250–510)
*p* value	*0.031* ^*∗*^	*<0.0001* ^*∗*^	*0.017* ^†^	*<0.0001* ^*∗*^	1.0^*∗*^	*0.08* ^†^

CDVA: corrected distance visual acuity; CRT: central retinal thickness; ERM: epiretinal membrane; RRD: rhegmatogenous retinal detachment. ^*∗*^Wilcoxon test; ^†^paired *t*-test.

**Table 3:** Baseline predictors of final visual outcome after 12 months after the first DEX injection.

Baseline measure	CDVA loss >1 line *n* (%)	CDVA gain or loss ≤1 line *n* (%)	CDVA gain >1 line *n* (%)	*p* value	OR (95% CI)^*∗*^
Baseline CDVA (log MAR ± SD)^†^	0.47 ± 0.34	0.50 ± 0.26	0.70 ± 0.30	0.001	1.485 (1.171–1.884)

Indication for surgery				0.046	1.168 (1.003–1.360)
ERM	5/46 (11)	6/46 (13)	35/46 (76)		
RRD	4/15 (27)	4/15 (27)	7/15 (46)		

Lens status				0.820	1.015 (0.890–1.159)
Phakic	3/27 (11)	5/27 (19)	19/27 (70)		
Pseudophakic	6/34 (18)	5/34 (15)	23/34 (67)		

Naïve status				0.853	0.946 (0.871–1.137)
Naïve	2/18 (11)	2/18 (11)	14/18 (78)		
Previously treated	7/43 (16)	8/43 (19)	28/43 (65)		

IRF/cysts				0.465	1.055 (0.915–1.216)
Absence of IRF/cysts	1/8 (12)	2/8 (25)	5/8 (63)		
Presence of IRF/cysts	7/53 (13)	8/53 (15)	38/53 (72)		

SRF				0.043	0.860 (0.743–0.995)
Absence of SRF	8/43 (19)	9/43 (21)	26/43 (60)		
Presence of SRF	0/18 (0)	1/18 (6)	17/18 (94)		

EZ continuity				0.209	1.094 (0.951–1.257)
Completely continuous	4/12 (33)	2/12 (17)	6/12 (50)		
Partially disrupted	3/31 (10)	3/31 (10)	25/31 (80)		
Completely disrupted	1/18 (6)	5/18 (28)	12/18 (66)		

Onset of PCME to surgery				0.827	0.985 (0.871–1.137)
<3 months	7/38 (18)	3/38 (8)	28/38 (74)		
>3 months	2/23 (9)	7/23 (30)	14/23 (61)		

Time between surgery and first DEX				0.982	0.999 (0.901–1.107)
<6 months	6/36 (17)	7/36 (19)	23/36 (64)		
>6 months	3/25 (12)	3/25 (12)	19/25 (76)		

CDVA: corrected distance visual acuity; CI: confidence interval; ERM: epiretinal membrane; RRD: rhegmatogenous retinal detachment; IRF: intraretinal fluid; SRF: subretinal fluid; EZ: ellipsoid zone; PCME: postoperative cystoid macular edema; DEX: dexamethasone intravitreal implant. ^*∗*^Odds ratio (OR) for a patient presenting with a gain of CDVA >1 line at 12 months when baseline measure is increased by 1 value. ^†^For every less line (+0.1 log MAR) of baseline BCVA, a patient was more likely to gain >1 line in CDVA at 12 months after initial DEX injection.

## Data Availability

The data used to support the findings of this study are available from the corresponding author upon request.
